# Grading of Endocervical Adenocarcinomas: Review of the Literature and Recommendations From the International Society of Gynecological Pathologists

**DOI:** 10.1097/PGP.0000000000000741

**Published:** 2021-02-09

**Authors:** Karen L. Talia, Esther Oliva, Joseph T. Rabban, Naveena Singh, Simona Stolnicu, W. Glenn McCluggage

**Affiliations:** Department of Pathology, Royal Women’s Hospital and VCS Foundation, Melbourne, Australia (K.L.T.); Department of Pathology, Massachusetts General Hospital, Boston, Massachusetts (E.O.); Pathology Department, University of California San Francisco, San Francisco, California (J.T.R.); Department of Pathology, University of Medicine, Pharmacy, Sciences and Technology of Targu Mures, Romania (S.S.); Department of Cellular Pathology, Barts Health NHS Trust, London (N.S.); Department of Pathology, Belfast Health and Social Care Trust, Belfast, UK, (W.G.M.C.)

**Keywords:** Endocervical adenocarcinoma, Grading, Pattern-based classification

## Abstract

There is a lack of consensus regarding the prognostic value of grading endocervical adenocarcinomas and currently, no universally applied, validated system for grading exists. Several grading schemes have been proposed, most incorporating an evaluation of tumor architecture and nuclear morphology and these are often based on the International Federation of Gynecology and Obstetrics (FIGO) system for endometrial endometrioid carcinoma, although some schemes modify the proportion of solid tumor required to separate grades 1 and 2 from 5% to 10%. In the absence of a validated system, we endorse this approach for most human papillomavirus–associated endocervical adenocarcinomas and, based on the available evidence, recommend that tumors with ≤10% solid growth be designated grade 1, 11% to 50% solid growth grade 2 and >50% solid growth grade 3. Tumors should be upgraded in the presence of marked nuclear atypia involving the majority (>50%) of the tumor. Grading is not recommended for human papillomavirus-independent adenocarcinomas, since no validated system has been suggested and most of these neoplasms exhibit intrinsically aggressive behavior regardless of their morphologic appearance. Importantly, grading should not be performed for gastric-type adenocarcinomas, particularly as these tumors may appear deceptively “low-grade” yet still exhibit aggressive behavior. Recently devised, validated and reproducible etiology and pattern-based tumor classification systems for endocervical adenocarcinomas appear to offer more effective risk stratification than tumor grading and, in the future, these systems may render the provision of a tumor grade redundant.

Significant advances in our understanding of endocervical adenocarcinoma and efforts to more effectively tailor treatment have resulted in revised tumor classification and pattern-based systems for these neoplasms in recent years; these are discussed in other reviews in this issue 1,2. The International Endocervical Criteria and Classification (IECC) system classifies tumors based on etiology and surrogate morphology into human papillomavirus (HPV) associated (HPVA) adenocarcinomas, characteristically associated with prominent apical mitoses and basal apoptoses, and non–HPV-associated (NHPVA) adenocarcinomas, with the latter generally being more aggressive with high stage at diagnosis and worse overall survival 3–5. For clarity, while the IECC system utilizes the terminology “HPVA” and “NHPVA” adenocarcinoma, hereafter we shall use the terminology adopted by the World Health Organisation in the recently published fifth edition of Tumours of Female genital organs 6, where cervical adenocarcinomas are designated “HPVA” and “HPV independent (HPVI).” The Silva Pattern Classification system, which applies only to HPVA adenocarcinomas, subdivides tumors into 3 prognostic groups based upon the presence and extent of destructive stromal invasion, nuclear grade and architecture, correlating with risk of lymph node metastasis and clinical outcome 7–9.

An aspect of the evaluation of cervical adenocarcinomas not formally addressed by either of these schemes, and which generally has achieved scant coverage in the literature, is tumor grading, including grading criteria and its clinical significance. This is an unresolved and controversial issue, with no consensus regarding the independent prognostic value of tumor grade, which is further compounded by the lack of a validated and universally applied grading system. Despite this, there is an expectation from clinicians that tumor grade will be included in the pathology report, irrespective of whether it will influence treatment. As an aside, similar comments pertain to cervical squamous cell carcinomas (SCCs) and this has been discussed in a recent review 10.

In this review, as part of the International Society of Gynecological Pathologists (ISGyP) project on endocervical adenocarcinomas, we examine the existing literature on grading of these tumors, focussing on the clinical relevance of this parameter, existing grading systems and current recommendations from pathology societies. We also explore the future relevance of grading in light of the aforementioned developments and their evolving impact on pathology reporting. Despite a lack of robust evidence, we provide recommendations for current-day pathology reporting, acknowledging that this is an area requiring further study and likely, in future, to undergo change.

## PROBLEMATIC ASPECTS OF GRADING

In the absence of a validated grading system for endocervical adenocarcinoma, practice varies among pathologists, as evidenced by responses from members surveyed for the ISGyP endocervical adenocarcinoma project 11. Of 151 respondents, 25.8% stated they did not report a grade for endocervical adenocarcinoma; 47.7% utilize the International Federation of Gynecology and Obstetrics (FIGO) grading system for endometrial endometrioid carcinoma and 26.5% provide a grade but use a different system. Generally, these alternative methods of grading constituted a poorly defined 3-tier stratification based upon the relative proportion of gland formation versus solid tumor and degree of nuclear atypia (eye-balling). Some respondents to the survey applied the Silva Pattern Classification in place of or as an adjunct to reporting tumor grade. The lack of a consensus approach is not the only problematic aspect of tumor grading. Even with rigid definitions and thresholds, grading is an inherently subjective process and in certain settings, such as when evaluating a small biopsy or where invasion is very limited in extent, may be of limited value. Furthermore, no grading system can be universally applied across all tumor types given the innate differences in behavior of the various types of endocervical adenocarcinoma. For example, gastric-type adenocarcinoma, the commonest HPVI adenocarcinoma, is more aggressive than HPVA adenocarcinoma, irrespective of the degree of differentiation 12. It follows that applying the FIGO grading system for endometrial carcinoma, or a similar system, to morphologically well-differentiated gastric-type adenocarcinomas may lead to under-appreciation of the risk of aggressive behavior and potentially result in under-treatment.

## PROGNOSTIC SIGNIFICANCE OF TUMOR GRADE AND IMPACT ON MANAGEMENT

The published literature examining the prognostic significance of tumor grade for endocervical adenocarcinoma reaches varied conclusions and meaningful comparison of the data is hampered by the diverse and sometimes undocumented grading systems used. Furthermore, in many studies details are not provided regarding the histologic types included; thus outcome data are potentially skewed by the admixture of HPVA adenocarcinoma and aggressive types of HPVI adenocarcinoma such as gastric-type 13–15. This is not surprising given that until relatively recently, endocervical adenocarcinomas were considered a fairly homogenous group of neoplasms which were largely HPVA with the exception of minimal deviation adenocarcinoma/adenoma malignum (now designated gastric-type adenocarcinoma), clear cell and mesonephric carcinomas.

A number of studies have examined cervical adenocarcinoma outcomes against a range of histologic parameters including tumor grade. Baalbergen et al. 16 examined 305 adenocarcinomas of mixed types using a 3-tier grading system combining architectural and cytologic features similar to that employed for endometrial endometrioid carcinoma but with cut-off points for the proportion of solid architecture set at ≤10% (grade 1), 11% to 50% (grade 2) and >50% (grade 3), with tumors upgraded for marked nuclear atypia. By multivariate analysis, they found FIGO stage, grade and lymph node metastasis to be significant independent predictors of 5-yr survival. Another study used the same grading system and examined 129 HPVA adenocarcinomas 17. On univariate analysis, tumor grade, nodal metastasis, lymphovascular space invasion (LVSI), parametrial involvement, depth of invasion, tumor size and tumor budding all correlated with survival, but on multivariate analysis, grade 3 histology was the only independent predictor of decreased disease-free and cancer-specific survival. Nola et al. 18 similarly used this grading system to evaluate 36 adenocarcinomas of mixed types (a small number of cases) and also applied a 3-tier nuclear grade. By univariate analysis, both architectural and nuclear grade were significant in terms of 5-yr survival but on multivariate analysis, nuclear grade was the only significant independent parameter with the 5-yr survival for tumors with grade 1 nuclei 80% and for grade 3 nuclei 30%. A further recent study examined 71 HPVA adenocarcinomas, aiming to elucidate what features, together with Silva pattern, predict aggressive behavior and lymph node status 19. A 3-tier system was devised to assign nuclear grade, with the authors finding that both grade 3 nuclei and necrotic luminal debris were predictive of pattern C invasion, LVSI and higher tumor stage 19. Finally, 2 large multicenter Surveillance, Epidemiology and End Results Program (SEER) population analyses of both SCCs and adenocarcinomas found tumor grade to be a significant independent prognostic variable for survival on multivariate analysis, correlating with risk of lymph node metastasis in one study 13,15; however, the grading systems used in these population-based studies were not detailed.

By comparison, a number of other studies have not found grade to have independent prognostic value. Khalil et al. 14 applied an unspecified 3-tier grading system to 350 adenocarcinomas of mixed types and, while tumor grade was significant in terms of patient survival on univariate analysis and correlated with the risk of lymph node metastasis, it was not a significant independent variable on multivariate analysis. Others have documented similar findings, with tumor grade losing its independent prognostic significance on multivariate analysis 20. More recently, a study examining clinical outcomes of 205 endocervical adenocarcinomas classified using IECC criteria found that tumor grade was not statistically significant in terms of patient survival in either HPVA adenocarcinomas, HPVI adenocarcinomas, or the entire cohort combined 4. This study used the endometrial endometrioid carcinoma FIGO grading system (with a 5% cut-off for proportion of solid elements between grades 1 and 2) and concluded that HPVA adenocarcinomas should not be assigned a grade but that the Silva Pattern Classification is more informative with respect to overall survival. For HPVI adenocarcinomas, there were no associations between histologically assessed features and clinical outcomes 4. Another study co-authored by one of us (S.S., currently under review) applied endometrial endometrioid carcinoma FIGO grading to 464 stage IB endocervical adenocarcinomas, most of which were grade 2. Interestingly, the frequency of grade 3 tumors increased with IB substage (11% in stage IB1, 16% in IB2 and 34% in IB3; *P*=0.00001), with larger tumors mostly conforming to Silva pattern C. A statistically significant association between high tumor grade and recurrence-free survival was also found but not with overall survival. While high tumor grade was a significant variable on univariate analysis when comparing IB substages, it did not carry prognostic significance on multivariate analysis.

Taken together, these studies provide some support for grading HPVA adenocarcinomas (but not HPVI adenocarcinomas, see below) using a grading system based on a combination of architectural and cytologic features, such as the FIGO grading system for endometrial endometrioid carcinomas or a modification of this (such as using a 10% rather than a 5% cut-off for the amount of solid architecture in distinguishing between grade 1 and 2). This is an area where clearly more studies are needed, including large prospective multicenter studies.

While most clinical guidelines state that tumor grade is an important element in pathologic tumor evaluation, it is not considered a major prognostic factor in endocervical adenocarcinomas and does not feature in treatment algorithms guiding patient management 21–23. Instead, primary management decisions are largely based on FIGO stage, tumor size, depth of invasion, lymph node status and presence or absence of LVSI. Similarly, guidelines, such as the “Sedlis Criteria,” which are used to guide adjuvant treatment decisions following radical hysterectomy, are based on depth of stromal invasion, LVSI and tumor size with grade not taken into account 21.

## EXISTING GUIDELINES FOR GRADING ENDOCERVICAL ADENOCARCINOMA

The International Collaboration on Cancer Reporting (ICCR) has developed, in conjunction with ISGyP, a structured cervical cancer reporting protocol with explanatory comments 24. This ICCR data set includes tumor grade as a noncore (recommended) rather than a core (required) data element for both SCC and adenocarcinoma; as discussed, this partly reflects the lack of a universally applied, validated grading system but also the conflicting data regarding its clinical significance 24. The ICCR data set cites the most commonly used approach, based on the FIGO system for grading endometrial endometrioid carcinomas, but does not specifically recommend its use. The most current Australasian and United Kingdom cervical cancer data sets adopt the same approach, although the United Kingdom Royal College of Pathologists dataset lists tumor grade as a core element 25,26. The latest College of American Pathologists (CAP) protocol 27 advocates a 3-tier grading system based on architecture (glandular or papillary vs. solid tumor growth) and nuclear features and states that most literature supports its prognostic value. Grade 1 tumors have a small component (not further quantified) of solid growth and mild to moderate atypia, grade 3 tumors have a predominantly solid pattern with severe nuclear atypia and grade 2 show intermediate features.

A number of published systems are cited by these authorities and most represent subtle variations of a similar 3-tier stratification based on the proportion of solid tumor growth and degree of nuclear atypia. The earliest system, proposed by Lawrence and colleagues in 2000, set thresholds for architectural grading at 10%, 11% to 50%, and >50% solid growth (grades 1–3, respectively) and stated that nuclei in grade 1 tumors are uniform, oval, and minimally stratified, those in grade 2 tumors are more rounded and irregular with micronucleoli and more frequent mitoses, while those in grade 3 tumors are large, irregular and pleomorphic. In addition to nuclear features and extent of solid architecture, grade 3 criteria included the following: occasional signet ring cells, high, and abnormal mitotic activity, associated pronounced desmoplasia and necrosis 28. Several subsequent publications concur with these numerical cut-off points for the proportion of solid tumor growth, also specifying upgrading for marked nuclear atypia 16–18. By comparison, Silverberg and Ioffe 29 advocate precise adherence to the endometrial carcinoma system, utilizing ≤5%, 6% to 50% and >50% cut-off points for the solid tumor component.

In 2002, Young and Clement 30 similarly advocated a 3-tier grading system but did not provide specific diagnostic criteria. They emphasized that endocervical adenocarcinomas are usually more cytologically atypical than architecturally matched endometrioid carcinomas and, despite well-developed glandular architecture, generally show at least moderate nuclear atypia and brisk mitotic activity, most often conforming to grade 2. They cautioned against undergrading, stating that a designation of grade 1 should be reserved for well-differentiated villoglandular adenocarcinomas exhibiting exclusively low-grade cytology. The various grading systems discussed are provided in more detail in Table 1.

**TABLE 1:** Published grading systems for endocervical adenocarcinoma

Source	Grading scheme
Lawrence et al. 28	Grade by architectural (% of solid growth, excluding squamous) and cytologic (nuclear) criteria Grade 1: well-differentiated (<10% solid growth) The tumor contains well-formed regular glands with papillae. The cells are elongate and columnar with uniform oval nuclei, show minimal stratification (fewer than 3 cell layers in thickness), mitotic figures infrequent Grade 2: moderately differentiated (11%–50% solid growth) The tumor contains complex glands with frequent bridging and cribriform formation. Solid areas are more common but make up less than half of the tumor. Nuclei are more rounded and irregular, micronucleoli are present, mitoses more frequent. Grade 3: poorly differentiated (>50% solid growth) The tumor contains sheets of malignant cells; few glands are discernible. The cells are large and irregular with pleomorphic nuclei. Occasional signet ring cells are present. Mitoses are abundant with abnormal forms. Desmoplasia is pronounced and necrosis common
Young and Clement 30	No specific criteria suggested. Avoid undergrading; although many tumors are uniformly gland-forming most show at least moderate nuclear atypia and brisk mitoses. Most endocervical carcinomas are grade 2 of 3 The diagnosis of well-differentiated villoglandular papillary adenocarcinoma should be reserved for lesions that are exclusively grade 1
Silverberg and Ioffe 29	Grading of any endocervical adenocarcinoma is according to the FIGO system for endometrial adenocarcinomas on the basis of the amount of solid component Grade 1: <5% solid architecture Grade 2: > 5%–50% solid architecture Grade 3: >50% solid architecture
Baalbergen et al. 16	Grade using architectural and nuclear features Grade 1: well-differentiated. >90% glandular and tubular architecture Grade 2: moderately differentiated. 50-90% glandular and tubular architecture Grade 3: poorly differentiated. <50% glandular and tubular architecture. Where nuclear atypia is marked the tumor is allocated to a less differentiated category. Clear cell carcinomas are not graded
Nola et al. 18	Architectural and nuclear grade are determined separately. Architectural grade is based on the proportion of solid growth of the nonsquamous component. Well-differentiated: <10% not forming glands or tubules Moderately differentiated: 10%–50% not forming glands or tubules Poorly differentiated: >50% not forming glands or tubules Nuclear grade is evaluated in the most atypical area. Grade 1: cells with oval nuclei without prominent nucleoli and with evenly dispersed chromatin Grade 2: Nuclear features in between Grades 1 and 3 Grade 3: cells with markedly enlarged nuclei displaying irregular coarse chromatin and prominent nucleoli. The presence of grade 3 nuclear features in most neoplastic cells in architecturally well and moderately differentiated tumors raises the architectural grade by 1
Rivera-Colon et al. 19	Nuclear grade is applied Grade 1 (low): uniform, elongate, hyperchromatic nuclei with no or mild chromatin clearing and inconspicuous nucleoli. Nuclear features resemble those in adenocarcinoma in situ. Grade 2 (intermediate): subtle areas with grade 3 nuclei in <50% of the tumor. Grade 3 (high): nuclear enlargement and pleomorphism, nuclear membrane irregularity, clumped chromatin with areas of chromatin clearing and nucleolar prominence
CAP guidelines 27	Grade 1: small component of solid growth and mild to moderate atypia Grade 2: intermediate between Grades 1 and 3 Grade 3: solid pattern with severe nuclear atypia Tumors with no or focal minimal differentiation are designated undifferentiated carcinomas and categorized as grade 4

## TUMOR TYPES NOT AMENABLE TO GRADING

Like endometrial adenocarcinomas, there is a small but important subset of endocervical adenocarcinomas that are intrinsically high-grade and for which grading does not apply; most, but not all, of these represent HPVI adenocarcinomas.

Of the HPVA adenocarcinomas, several variants with inherently aggressive behavior are unsuited to grading and are automatically considered high-grade. Cervical adenocarcinoma with a micropapillary component is a recently recognized variant of HPVA adenocarcinoma with a poor prognosis. In the largest study of these tumors, all 44 cases exhibited LVSI, 41 of 44 (93%) had lymph node metastases and patient outcome did not vary with the extent of micropapillary growth 31. Similarly, some variants of HPVA mucinous adenocarcinoma, such as signet ring type and invasive stratified mucinous carcinoma (ISMC), have a poor prognosis and are not amenable to grading 32. Given the adverse outcome of these variants, tumors with a micropapillary, signet ring or ISMC component (regardless of the percentage of this component if admixed with usual HPVA adenocarcinoma) should be considered high-grade. It should also be noted that some usual HPVA adenocarcinomas have a minor or even predominant papillary or villoglandular architecture and this is different from a micropapillary architecture which is characterized by small, tightly cohesive papillary groups of neoplastic cells with eosinophilic cytoplasm and atypical nuclei, typically surrounded by clear spaces resembling vascular channels 1,31.

Gastric-type adenocarcinoma, representing the most common HPVI adenocarcinoma 3, is an aggressive tumor type with poor prognosis irrespective of the degree of differentiation and should be considered high-grade regardless of morphology. Karamurzin et al. 12 compared the outcome of 38 patients with gastric-type adenocarcinoma to that of 139 HPVA adenocarcinomas and demonstrated a disease-specific 5-yr survival of 42% for gastric-type adenocarcinoma compared with 91% for HPVA adenocarcinoma. Gastric-type adenocarcinomas presented at a more advanced stage and extremely well-differentiated tumors, previously termed minimal deviation adenocarcinoma or adenoma malignum, showed no survival advantage over poorly differentiated neoplasms. Another recent study of 328 endocervical adenocarcinomas similarly found worse disease-free and overall survival for gastric-type adenocarcinoma 33. Recurrence rate in gastric-type adenocarcinomas was 40% compared with 14.6% for HPVA adenocarcinomas and while response rates to chemotherapy were similar (36.8% vs. 32%), gastric-type adenocarcinoma was significantly more resistant to radiotherapy. Compared with HPVA adenocarcinomas, gastric-type adenocarcinomas were significantly associated with tumor diameter >4 cm, deep stromal invasion, LVSI, parametrial invasion, ovarian and pelvic lymph node metastasis and positive peritoneal cytology. No correlation was found between these morphologic predictors of poor outcome and the degree of tumor differentiation 33.

Clear cell carcinoma (CCC), representing 20% of HPVI adenocarcinomas and ~3% of all endocervical adenocarcinomas 3,34, resembles its counterparts in the endometrium and ovary, where it is automatically considered a high-grade tumor. CCC is a morphologically distinct tumor type and, while the prognosis may be no worse than that for HPVA adenocarcinoma, currently no grading system is recommended for this tumor (in the cervix or elsewhere). In the cervix, limited data suggest that CCC has a similar prognosis to HPVA adenocarcinoma when controlled for stage 34–37 and further studies are required to establish clinical outcomes for this tumor relative to HPVA adenocarcinoma. Mesonephric adenocarcinoma, comprising <1% of all cervical adenocarcinomas and 2% of HPVI adenocarcinomas 3,34,38, is another rare tumor for which no grading system exists.

Grading is also not recommended when an endocervical adenocarcinoma is admixed with a component of neuroendocrine carcinoma, either small cell or large cell type. Neuroendocrine carcinomas are automatically considered high-grade and this applies irrespective of the percentage of the neuroendocrine component, as even a small component of neuroendocrine carcinoma may be associated with adverse outcome 39. Such tumors should be classified as “adenocarcinoma admixed with neuroendocrine carcinoma” 6 and the individual tumor types and their percentages should be provided in the pathology report.

## SILVA PATTERN CLASSIFICATION: IS GRADING STILL NEEDED?

Recently it has been suggested that a combination of the morphology-based Silva Pattern Classification and IECC tumor type could form the basis for prognostic stratification of cervical adenocarcinomas, with the Silva Classification acting as a surrogate for tumor grade 4,34. The Silva Pattern Classification (discussed in detail in another review in this issue) quantifies the risk of nodal metastasis in HPVA adenocarcinomas and, although based on pattern of growth and invasion, also incorporates assessment of tumor architecture and nuclear grade. Pattern A and B tumors are architecturally well to moderately differentiated and exhibit no solid growth, whereas pattern C tumors may be architecturally high-grade with solid, confluent growth. Nuclear grade is considered only for pattern A tumors which should not be diagnosed in the presence of high-grade cytologic atypia; nuclear grade is disregarded in pattern C 7–9,40. Importantly, the reproducibility and prognostic value of the Silva and IECC classifications has been validated 4,5,7–9, whereas, as discussed, data regarding the value of tumor grade are conflicting and in most cases, grade does not impact on clinical decision-making. Given the many shortcomings of tumor grading, it is conceivable that in the future, evaluation of tumor grade may become redundant.

## CONCLUSIONS

As we transition to a new approach to reporting endocervical adenocarcinomas, it is uncertain what place tumor grade will have in future risk algorithms. The outcome of international collaborative efforts, such as the current ISGyP endocervical adenocarcinoma project, will hopefully help to clarify this issue. However, until such time as this question is resolved, it is important that a uniform approach to grading is currently adopted, particularly as this will enable prospective data collection and facilitate further study. In the absence of a properly validated grading system, we recommend an approach based on a combination of tumor architecture and cytology, recognizing that data in support of this is limited. As discussed above, there is some evidence that such systems may be of prognostic value and we recommend employing the most commonly used cut-offs for solid architecture set at ≤10% (grade 1), 11% to 50% (grade 2), and >50% (grade 3). Tumors can be upgraded in the presence of marked nuclear atypia as in the FIGO grading system for endometrial endometrioid carcinomas 41. When applying this system to endometrial endometrioid carcinomas, ISGyP has recommended that severe nuclear atypia should be present in the majority of cells (>50%) to upgrade a grade 1 or 2 endometrioid carcinoma 41 and a similar approach could be used when grading endocervical adenocarcinomas, although there is little literature regarding this issue. ISGyP has also recommended that a confluent microglandular/ microacinar pattern be regarded as a solid component when grading endometrial endometrioid carcinomas 41 and we endorse this approach for those rare HPVA cervical adenocarcinomas where this pattern is present. We recommend that grading be applied predominantly to HPVA adenocarcinomas, excluding variants with a micropapillary, signet ring or ISMC component, as these are automatically considered high-grade. Endometrioid carcinomas are an extremely rare variant of primary endocervical adenocarcinoma (discussed in another issue in this review 2) which are HPVI and could also be graded using this system.

**FIG. 1:**
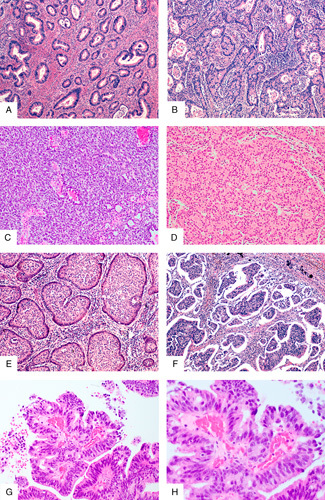
Grading of human papillomavirus associated (HPVA) adenocarcinoma. Grade 1 HPVA adenocarcinoma exhibits ≤10% solid growth (A), grade 2 10% to 50% solid growth (B) and grade 3 >50% solid growth (C); in this example the tumor is almost completely solid with only occasional poorly formed glands. A confluent microglandular/microacinar pattern (D) is regarded as a solid component for grading purposes. Some variants of HPVA adenocarcinoma with intrinsically aggressive behavior such as invasive stratified mucinous carcinoma (E) and micropapillary carcinoma (F) should not be graded. Significant nuclear atypia in HPVA adenocarcinoma (G, H); if such nuclei are present in the majority of tumor cells (>50%) the tumor can be upgrade from grade 1 to 2 or grade 2 to 3.

Aspects of grading of HPVA adenocarcinomas are illustrated in Figure 1.

## RECOMMENDATIONS

1. HPVA endocervical adenocarcinomas (with some exceptions) should be graded using a combination of architecture and cytology.
2. HPVA endocervical adenocarcinomas with ≤10% solid growth are grade 1, 11% to 50% solid growth grade 2 and >50% solid growth grade 3. Tumors can be upgraded in the presence of marked nuclear atypia involving >50% of the tumor.
3. HPVI adenocarcinomas should not be graded; in particular, gastric-type adenocarcinomas should not be graded but considered high-grade regardless of morphology.
4. Endocervical adenocarcinoma admixed with neuroendocrine carcinoma should not be graded but considered high-grade regardless of morphology.

## References

[1] Alvarado-Cabrero I Parra-Herran C Stolnicu S. International Society of Gynecological Pathologists (ISGyP) Endocervical Adenocarcinoma Project: distinction between In-Situ and Invasive Endocervical Adenocarcinoma and Pattern-based Classification (Silva System). Int J Gynecol Pathol 2020. In press. 10.1097/PGP.0000000000000735 PMC7969170 33570863

[2] Stolnicu S Park KJ Kiyokawa T. Endocervical adenocarcinoma: contemporary review of tumour typing and recommendations from the International Society of Gynecological Pathologists. Int J Gynecol Pathol 2020. In press. 10.1097/PGP.0000000000000751 PMC7888380 33570865

[3] Stolnicu S Barsan I Hoang L. International Endocervical Adenocarcinoma Criteria and Classification (IECC): a new pathogenetic classification for invasive adenocarcinomas of the endocervix. Am J Surg Pathol 2018;42:214–26. 29135516 10.1097/PAS.0000000000000986 PMC5762258

[4] Stolnicu S Hoang L Chiu D. Clinical outcomes of HPV-associated and unassociated endocervical adenocarcinomas classified by the International Endocervical Adenocarcinoma Criteria and Classification (IECC). Am J Surg Pathol 2019;43:466–74. 30720532 10.1097/PAS.0000000000001224 PMC6417947

[5] Hodgson A Olkhov-Mitsel E Howitt BE. International Adenocarcinoma Criteria and Classification (IECC): correlation with adverse clinicopathological features and patient outcome. J Clin Pathol 2019;72:347–53. 30679193 10.1136/jclinpath-2018-205632

[6] WHO Classification of Tumours Editorial Board. Female Genital Tumours (WHO Classification of Tumours Series. 5th ed. Vol. 4. Lyon, France): International Agency for Research on Cancer; 2020.

[7] Diaz De Vivar A Roma AA Park KJ. Invasive endocervical adenocarcinoma: proposal for a new pattern-based classification system with significant clinical implications: a multi-institutional study. Int J Gynecol Pathol 2013;32:592–601. 24071876 10.1097/PGP.0b013e31829952c6

[8] Roma AA Diaz De Vivar A Park KY. Invasive endocervical adenocarcinoma: a new pattern-based classification system with important clinical significance. Am J Surg Pathol 2015;39:667–72. 25724003 10.1097/PAS.0000000000000402

[9] Rutgers JK Roma AA Park KJ. Pattern classification of endocervical adenocarcinoma: reproducibility and review of criteria. Mod Pathol 2016;29:1083–94. 27255163 10.1038/modpathol.2016.94 PMC5506840

[10] McCluggage WG. Towards developing a meaningful grading system for cervical squamous cell carcinoma. J Path Clin Res 2018;4:81–5. 29665326 10.1002/cjp2.98 PMC5903690

[11] McCluggage WG Rabban JT Singh N. Survey results on pathological aspects of cervical adenocarcinoma by the International Society of Gynecological Pathologists (ISGyP). Int J Gynecol Pathol 2020. In press. 10.1097/PGP.0000000000000744 PMC7969156 33687170

[12] Karamurzin Y Takako K Parkash V. Gastric-type endocervical adenocarcinoma: An aggressive tumour with unusual metastatic patterns and poor prognosis. Am J Surg Pathol 2015;39:1449–57. 26457350 10.1097/PAS.0000000000000532 PMC4976691

[13] Vinh-Hung V Bourgain C Vlastos G. Prognostic value of histopathology and trends in cervical cancer: a SEER population study. BMC Cancer 2007;7:164. 17718897 10.1186/1471-2407-7-164 PMC1994954

[14] Khalil J Bellefqih S Afif M. Prognostic factors affecting cervical adenocarcinoma: 10 years experience in a single institution. Arch Gynecol Obstet 2015;292:915–21. 25851496 10.1007/s00404-015-3701-6

[15] Macdonald OK Chen J Dodson M. Prognostic significance of histology and positive lymph node involvement following radical hysterectomy in carcinoma of the cervix. Am J Clin Oncol 2009;32:411–6. 19451800 10.1097/COC.0b013e31819142dc

[16] Baalbergen A Ewing-Graham PC Hop WCJ. Prognostic factors in adenocarcinoma of the uterine cervix. Gynecol Oncol 2004;92:262–7. 14751169 10.1016/j.ygyno.2003.09.001

[17] Satabongkoch N Khunamornpong S Pongsuvareeyakul T. Prognostic value of tumor budding in early-stage cervical adenocarcinomas. Asian Pac J Cancer Prev 2017;18:1717–22. 28670894 10.22034/APJCP.2017.18.6.1717 PMC6373798

[18] Nola M Tomicic I Dotlic S. Adenocarcinoma of uterine cervix—prognostic significance of clinicopathologic parameters. Croat Med J 2005;46:397–403. 15861518

[19] Rivera-Colon G Chen H Niu S. Cervical adenocarcinoma: histopathologic features from biopsies to predict tumour behaviour. Am J Surg Pathol 2020;44:247–54. 31567190 10.1097/PAS.0000000000001379

[20] Chargui R Damak T Khomsi F. Prognostic factors and clinicopathologic characteristics of invasive adenocarcinoma of the uterine cervix. Am J Obstet Gynecol 2006;194:43–48. 16389008 10.1016/j.ajog.2005.06.029

[21] Koh WJ Aby-Rustum NR Bean S. NCCN clinical practice guidelines in oncology. Cervical Cancer, version 3. 2019. J Natl Comp Canc Netw 2019;17:64–84. 10.6004/jnccn.2019.0001 30659131

[22] Marth C Landoni F Mahner S. Cervical cancer: ESMO Clinical Practice Guidelines for diagnosis, treatment and follow-up. Ann Oncol 2017;28(suppl 4):iv72–83. 28881916 10.1093/annonc/mdx220

[23] Cibula D Potter R Planchamp F. The European Society of Gynaecological Oncology/European Society for Radiotherapy and Oncology/European Society of Pathology Guidelines for the management of patients with cervical cancer. Int J Gynecol Cancer 2018;28:641–55. 29688967 10.1097/IGC.0000000000001216

[24] McCluggage WG Judge MJ Alvarado-Cabrero I. Data set for the reporting of carcinomas of the cervix: recommendations from the International Collaboration on Cancer Reporting (ICCR). Int J Gynecol Pathol 2017;37:1–24. 10.1097/PGP.0000000000000412 28700433

[25] Royal College of Pathologists of Australasia. Structured reporting protocol for excisions and colposcopic biopsies performed for the diagnosis and treatment of pre-invasive cervical neoplasa (1st ed), (version 1.0). 2017. Available at: https://www.rcpa.edu.au/getattachment/9ed056b7-6bcc-4885-a243-925053302e3b/Protocol-Cervical-pre-neoplasia.aspx. Accessed March 2017.

[26] Royal College of Pathologists. Dataset for histological reporting of cervical neoplasia (3rd ed). 2011. Available at: https://www.rcpath.org/uploads/assets/eb26fb88-3db6-417b-97ee6338ef54dc79/g071cervicaldatasetapril11.pdf. Accessed March 21, 2011.

[27] College of American Pathologists. Protocol for the examination of resection specimens from patients with primary carcinoma of the uterine cervix. 2020. Available at: https://documents.cap.org/protocols/cp-femalereproductive-uterinecervix-resection-20-4300.pdf. Accessed February 2020.

[28] Lawrence WD Abdul-Karim FW Crum C. Recommendations for the reporting of surgical specimens containing uterine cervical neoplasms. Mod Pathol 2000;13:1029–33. 11007044 10.1038/modpathol.3880186

[29] Silverberg S Ioffe O. Pathology of cervical cancer. Cancer J 2003;9:335–47. 14690308 10.1097/00130404-200309000-00003

[30] Young RH Clement PB. Endocervical adenocarcinoma and its variants: their morphology and differential diagnosis. Histopathology 2002;41:185–207. 12207781 10.1046/j.1365-2559.2002.01462.x

[31] Alverado-Cabrero I McCluggage WG Estevez-Castro R. Micropapillary cervical adenocarcinoma: a clinicopathologic study of 44 cases. Am J Surg Pathol 2019;43:802–9. 30864975 10.1097/PAS.0000000000001245 PMC8258798

[32] Horn LC Handzel R Borte G. Invasive stratified mucin-producing carcinoma (i-SMILE) of the uterine cervix: report of a case series and review of the literature indicating poor prognostic subtype of cervical adenocarcinoma. J Cancer Res Clin Oncol 2019;145:2573–82. 31385027 10.1007/s00432-019-02991-3 PMC11810250

[33] Nishio S Mikami Y Tokunaga H. Analysis of gastric-type mucinous carcinoma of the uterine cervix—an aggressive tumor with a poor prognosis: a multi-institutional study. Gynecol Oncol 2019;153:13–9. 30709650 10.1016/j.ygyno.2019.01.022

[34] Park KJ. Cervical adenocarcinoma: Integration of HPV status, pattern of invasion, morphology and molecular markers into classification. Histopathology 2020;76:112–27. 31846527 10.1111/his.13995

[35] Wang D Zhao C Li F. Primary clear cell adenocarcinoma of the cervix: a clinical analysis of 18 cases without exposure to diethlystilbestrol. Obstet Gynecol Int 2019;2019:9465375. 31049066 10.1155/2019/9465375 PMC6458873

[36] Reich O Tamussino K Lahousen M. Clear cell carcinoma of the uterine cervix: pathology and prognosis in surgically treated stage IB-IIB disease in women not exposed in utero to diethylstilbestrol. Gynecol Oncol 2000;76:331–5. 10684706 10.1006/gyno.1999.5700

[37] Jiang X Jin Y Li Y. Clear cell carcinoma of the uterine cervix: clinical characteristics and feasibility of fertility-preserving treatment. Onco Targets Ther 2014;7:111–6. 24470762 10.2147/OTT.S53204 PMC3891640

[38] Silver SA Devouassouz-Shisheboran M Mezzetti TP. Mesonephric adenocarcinomas of the uterine cervix: a study of 11 cases with immunohistochemical findings. Am J Surg Pathol 2001;25:379–87. 11224609 10.1097/00000478-200103000-00013

[39] Horn LC Hentschel B Bilek K. Mixed small cell carcinomas of the uterine cervix: prognostic impact of focal neuroendocrine differentiation but not of Ki-67 labeling index. Ann Diagn Pathol 2006;10:140–3. 16730307 10.1016/j.anndiagpath.2005.07.019

[40] Park KJ Roma AA. Pattern based classification of endocervical adenocarcinoma: a review. Pathology 2018;50:134–40. 29241973 10.1016/j.pathol.2017.09.011 PMC7478857

[41] Soslow RA Tornos C Park KJ. Endometrial carcinoma diagnosis: Use of FIGO grading and genomic subcategories in clinical practice: Recommendations of the International Society of Gynecological Pathologists. Int J Gynecol Pathol 2019;38:S64–S74. 30550484 10.1097/PGP.0000000000000518 PMC6295928

